# MIER1 (mesoderm induction early response 1 homolog (Xenopus laevis))

**DOI:** 10.4267/2042/46943

**Published:** 2012-03-30

**Authors:** Laura L Gillespie, Gary D Paterno

**Affiliations:** Terry Fox Cancer Research Labs, Division of Biomedical Sciences, Faculty of Medicine, Memorial University, St John’s, NL, Canada (LLG, GDP)

## Identity

**Other names:** ER1, Er1, KIAA1610, MGC150641, MGC131940, MGC150640, MI-ER1, hMI-ER1, RP5-944N15.1, DKFZp781G0451

**HGNC (Hugo):** MIER1

**Location:** 1p31.3

### 

#### Note

MIER1 was identified by differential display as an immediate-early gene activated during fibroblast growth factor (FGF) induction of mesoderm differentiation in Xenopus laevis.

## DNA/RNA

**Figure:**
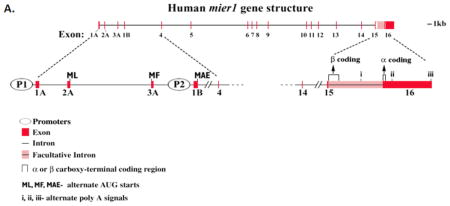
A. Schematic illustrating the exon-intron organization of the human MIER1 gene Exons are shown as red bars/vertical lines and introns as horizontal lines; exon numbers are indicated below each schematic. The light red bar indicates the facultative intron 16 and the position of the alpha and beta carboxy-terminal coding regions are indicated. Note that the beta coding region is located within the facultative intron. The three alternate starts of translation, ML-, MF- and MAE- are indicated as are the three polyadenylation signals (PAS): i, ii and iii.

**Figure:**
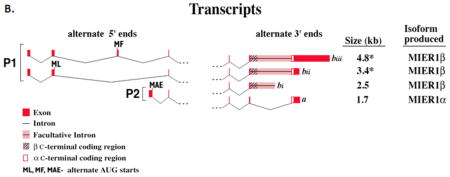
B. Schematic illustrating the variant 5′ and 3′ ends of human MIER1 transcripts Alternate 5′ ends are generated from differential promoter usage (P1 or P2) or alternate inclusion of exon 3A. This leads to three alternate starts of translation, indicated as ML-, MF- and MAE-, and produces three distinct amino termini. The four variant 3′ ends, a, bi, bii and biii, produced by alternative splicing or alternate PAS usage, result in transcripts readily distinguished by size (1.7 kb, 2.5 kb, 3.4 kb and 4.8 kb, respectively) on a Northern blot. It should be noted that three of the variant 3′ ends, bi, bii and biii encode the same protein sequence and differ only in their untranslated region. * indicates beta encoding transcript that contains the alpha exon in its 3′UTR. The locations of the alpha and beta carboxy-terminal coding regions and PAS i, ii and iii are indicated. The combination of three possible 5′ ends with four possible 3′ ends gives rise to 12 distinct transcripts, but only 6 distinct protein isoforms. In most adult tissues, the most abundant transcript is 4.8 kb. Additional transcripts have been reported in Ensembl.

### Description

63 kb gene; 2 promoters controlling 2 distinct transcriptional start sites; 17 exons; intron 16 is facultative; 3 polyadenylation sites.

## Protein

### Description

The six human MIER1 isoforms: M-3A-alpha (457 aa), M-3A-beta (536 aa), ML-alpha (432 aa), ML-beta (511 aa), MAE-alpha (433 aa), and MAE-beta (512 aa), range in predicted molecular size from 47.5 kDa-59 kDa; however all isoforms migrate slower than predicted on SDS-PAGE, with calculated molecular sizes ranging 78 kDa-90 kDa.

### Expression

MIER1beta protein is expressed ubiquitously, while MIER1alpha protein is expressed mainly in a subset of endocrine organs and endocrine responsive tissues, including the pancreatic islets, adrenal glands, testis, ovary, hypothalamus, pituitary, parafollicular cells of the thyroid and mammary ductal epithelium.

### Localisation

MIER1beta is nuclear in all adult cell types but is retained in the cytoplasm of the pre-gastrula Xenopus embryo. MIER1alpha is cytoplasmic in most cell types, but localized in the nucleus in normal mammary ductal epithelium. During progression to invasive breast carcinoma, its subcellular localization shifts from nuclear to exclusively cytoplasmic.

### Function

MIER1alpha and beta function in transcriptional repression by at least two distinct mechanisms: recruitment and regulation of chromatin modifying enzymes, including HDAC1, HDAC2, CBP and G9a; interaction with transcription factors, such as Sp1 and ERalpha, to repress transcription of their respective target genes. MIER1alpha inhibits estrogen-stimulated anchorage-independent growth of breast carcinoma cells.

### Homology

The MIER1 gene family contains two other members, MIER2 and MIER3. The MIER1 gene is conserved in chimpanzee, dog, cow, mouse, rat, chicken, frog, zebrafish, fruit fly, and C. elegans.

**Figure:**
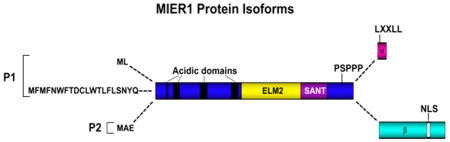
Schematic illustrating the common internal domains of the MIER1 isoforms and the variant amino- (N-) and carboxy- (C-) termini Transcription from the P1 promoter produces proteins that either begin with M-L- or with the sequence encoded by exon 3A (MFMFNWFTDCLWTLFLSNYQ). Transcription from the P2 promoter produces a protein that begins with M-A-E-. The variant N-termini of the MIER1 isoforms are followed by common internal sequence containing several distinct domains: acidic, which function in transcriptional activation (Paterno et al., 1997); ELM2, responsible for recruitment of HDAC1 (Ding et al., 2003); SANT, which interacts with Sp1 (Ding et al., 2004) and PSPPP, which is required for MIER1 activity in the Xenopus embryo (Teplitsky et al., 2003). The two alternate C-termini, alpha and beta, result from removal or inclusion and read-through of intron 16, respectively. The alpha C-terminus contains a classic LXXLL motif for interaction with nuclear receptors; the beta C-terminus contains a nuclear localization signal (NLS).

## Implicated in

### Breast cancer

#### Note

Initial studies showed that total MIER1 mRNA levels were increased in breast carcinoma cell lines and tumour samples (Paterno et al., 1998); in a more recent study, no consistent difference in MIER1alpha protein expression levels between normal breast and tumour samples was detected (McCarthy et al., 2008). Immunohistochemical analysis of patient biopsies revealed that MIER1alpha protein is expressed primarily in ductal epithelial cells in normal breast tissue, with little or no expression in the surrounding stroma; in breast carcinoma samples, its expression is restricted to tumour cells. While there is no difference in expression levels, the subcellular localization of MIER1alpha changes dramatically during tumour progression: MIER1alpha is nuclear in 75% of normal breast samples and in 77% of hyperplasia, but in breast carcinoma, only 51% of ductal carcinoma in situ, 25% of invasive lobular carcinoma and 4% of invasive ductal carcinoma contained nuclear MIER1alpha (McCarthy et al., 2008). This shift from nuclear to cytoplasmic localization of MIER1alpha during breast cancer progression suggests that loss of nuclear MIER1alpha contributes to the development of invasive breast carcinoma. MIER1alpha inhibits ERalpha transcriptional activity and overexpression of MIER1alpha in breast carcinoma cells inhibits estrogen-stimulated anchorage-independent growth (McCarthy et al., 2008).
